# Temporal Trends in Multimorbidity Patterns in Patients With Atrial Fibrillation—A Nationwide Cohort Study

**DOI:** 10.1111/eci.70253

**Published:** 2026-08-21

**Authors:** Sanni Reinilä, Niklas Kiuru, Ville Langén, Jussi Jaakkola, Olli Halminen, Jukka Putaala, Ossi Lehtonen, Pirjo Mustonen, Jari Haukka, Miika Linna, Juha Hartikainen, K. E. Juhani Airaksinen, Mika Lehto, Gregory Y. H. Lip, Konsta Teppo

**Affiliations:** ^1^ Department of Internal Medicine University of Turku Turku Finland; ^2^ Division of Medicine Turku University Hospital and University of Turku Turku Finland; ^3^ Heart Center Turku University Hospital, and University of Turku Turku Finland; ^4^ Heart Unit Satakunta Central Hospital Pori Finland; ^5^ Department of Health and Social Management University of Eastern Finland Kuopio Finland; ^6^ Neurology, Helsinki University Hospital, and University of Helsinki Helsinki Finland; ^7^ Department of Industrial Engineering and Management Aalto University Espoo Finland; ^8^ University of Helsinki Helsinki Finland; ^9^ University of Eastern Finland Kuopio Finland; ^10^ Heart Center Kuopio University Hospital Kuopio Finland; ^11^ Jorvi Hospital, HUS Helsinki University Hospital Espoo Finland; ^12^ Liverpool Centre for Cardiovascular Science at University of Liverpool, Liverpool John Moores University and Liverpool Heart & Chest Hospital Liverpool UK; ^13^ Department of Clinical Medicine Aalborg University, Aalborg University Hospital Aalborg Denmark; ^14^ Department of Cardiology, Lipidology and Internal Medicine With Intensive Coronary Care Unit Medical University of Bialystok Bialystok Poland

**Keywords:** atrial fibrillation, cardiovascular diseases, comorbidity groups, multimorbidity

## Abstract

**Background:**

The prevalence of both atrial fibrillation (AF) and multimorbidity is increasing with population aging. However, reliable and comprehensive data on temporal trends in multimorbidity among patients with AF are lacking.

**Methods:**

The nationwide Finnish AntiCoagulation in Atrial Fibrillation (FinACAF) cohort study includes all patients with incident AF between 2009 and 2018. Data on comorbidities were obtained from national healthcare, reimbursement and prescription registers using a fixed 5‐year look‐back period. A previously established comorbidity categorisation was applied to characterise multimorbidity burden. Temporal trends in the prevalence of common comorbidities and in the number of distinct diagnosis groups at the time of new‐onset AF were calculated.

**Results:**

Overall, 85% of 196,536 individuals with new onset AF had diagnoses from ≥ 2 comorbidity groups and 28% from ≥ 6 comorbidity groups. Mean number of different groups increased from 3.9 to 4.2 between 2009 and 2018. Although adjustment for age attenuated the association, the increase in multimorbidity over time remained significant after adjustment for age and sex. Multimorbidity was more common in women than in men (4.2 vs. 3.9) and increased with age. Hypertensive diseases, obesity and other metabolic diseases, diabetes and other cardiovascular diseases were most prevalent. The prevalence of ischaemic heart disease and other diseases of the heart and pulmonary circulation declined during the study period.

**Conclusions:**

Multimorbidity is highly prevalent and increasing among patients with AF, highlighting the importance of a holistic management approach.

## Graphical abstract

Overall, 85% of 196,536 patients with new onset atrial fibrillation had diagnoses from ≥ 2 comorbidity groups and 28% from ≥ 6 comorbidity groups. The mean number of different groups increased from 3.9 to 4.2 between 2009 and 2018. Multimorbidity was more common in women and increased with age. Hypertensive diseases, obesity and other metabolic diseases, diabetes and other cardiovascular diseases were most prevalent. The prevalence of ischaemic heart disease and other diseases of the heart and pulmonary circulation declined within the study period.

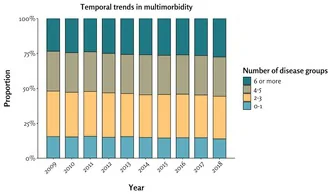

**Abbreviations**
AF: atrial fibrillation
ATC: anatomic therapeutic chemical
AvoHILMO: Register of Primary Health Care
CHA₂DS₂VA/CHA₂DSVASC: scoring system used for estimating ischaemic stroke risk in patients with atrial fibrillation (Congestive heart failure, Hypertension, Age ≥ 75 years, Diabetes mellitus, Stroke/TIA/thromboembolism in the past, Vascular disease, Age 65–74 years, Sex)
FinACAF: The Finnish AntiCoagulation in Atrial Fibrillation study
HILMO: Care Register for Specialised Health Care
ICD‐10: International Classification of Diseases, 10th Revision
ICPC‐2: International Classification of Primary Care, 2nd Edition
KELA: National Reimbursement Register

## 1 Introduction

Atrial fibrillation (AF) is the most common long‐standing arrhythmia, affecting approximately 5% of adults and as many as one in four individuals over the age of 75 [1]. The prevalence of AF is estimated to double over the next four decades, driven by increased longevity, enhanced detection of undiagnosed AF and improved survival from other cardiac conditions [2]. Concurrently, population aging is accompanied by a rising prevalence of numerous other chronic diseases.

Multimorbidity, defined by the National Institute for Health and Care Excellence as the coexistence of two or more long‐term conditions, is associated with functional decline, greater health care utilisation, elevated risk of adverse drug effects, reduced quality of life, polypharmacy and increased mortality [3, 4, 5, 6, 7]. Multimorbidity is highly prevalent, affecting approximately 80% of adults aged 65 years and older [8], and presents several well‐recognised challenges, such as fragmented care pathways, insufficiently tailored guidelines and a limited evidence base for patients with multiple conditions [9, 10].

Methods for assessing multimorbidity differ considerably across studies [11, 12], reflecting the ongoing challenge of establishing a consistent definition. This likely contributes to the inability of clinical practice guidelines for individual diseases to address the realities of multimorbidity [13]. A central question is whether a simple count of conditions provides a sufficient basis for healthcare policy. To address this limitation, more granular methods using disease and medication groups have been developed [12].

Disease grouping enables a more comprehensive representation of multimorbidity in electronic health records, overcoming challenges posed by complex or inconsistent diagnosis coding. By identifying patterns of co‐occurring diseases, grouping methods provide valuable insights for prognosis, targeted interventions, resource allocation and the underlying biological and social determinants of multimorbidity [8, 11, 12, 14, 15].

Patients with AF frequently present with additional comorbidities, partly due to shared pathophysiological mechanisms, such as those associated with metabolic diseases [15, 16, 17]. Although effective anticoagulation therapy has markedly improved stroke prevention in patients with AF, a substantial residual risk of adverse outcomes and persistent symptom burden remains [17, 18, 19, 20, 21, 22, 23, 24, 25]. The diagnosis of AF often prompts patients to seek medical attention, providing an opportunity to identify and manage otherwise silent or undiagnosed risk factors and comorbid conditions. Given the high burden of comorbidities in patients with AF, a holistic, integrated management approach has been shown to improve outcomes and is strongly endorsed by contemporary international AF guidelines [26, 27].

Although multimorbidity in AF is well recognised, few studies provide reliable prevalence estimates in unselected patient cohorts or track trends over time. These data are crucial for healthcare planning, for projecting the future burden of AF, and for guiding the development of guidelines that reflect the complexity of AF patients. To address this knowledge gap, we conducted a nationwide cohort study to characterise multimorbidity profiles and temporal trends in patients with AF.

## 2 Methods

### 2.1 Study Cohort

The Finnish AntiCoagulation in Atrial Fibrillation (FinACAF) study (ClinicalTrials Identifier: NCT04645537; ENCePP Identifier: EUPAS29845) is a nationwide retrospective cohort study that includes patients diagnosed with AF at all levels of care in Finland from 2004 to 2018. The construction of the cohort is more thoroughly described elsewhere [28]. Patients were identified using all available national healthcare registers, including hospitalisations and outpatient specialist visits (HILMO), primary healthcare (AvoHILMO) and the National Reimbursement Register maintained by the Social Insurance Institute (KELA). The cohort inclusion criterion was an International Classification of Diseases, 10th Revision (ICD‐10), diagnosis code of I48, encompassing AF and atrial flutter, collectively referred to as AF, recorded between 2004 and 2018. The exclusion criteria were permanent emigration before 31 December 2018 and age below 20 years at AF diagnosis.

The present sub‐study was conducted within a cohort of patients with incident AF from 2007 to 2018, originally established in earlier studies of the FinACAF cohort [29, 30, 31]. The current study included only patients with a first AF recording in 2009 or later, allowing for the collection of comorbidity data using a fixed 5‐year look‐back period prior to AF onset. Baseline information and comorbidities were collected from the hospital care register (HILMO) and National Reimbursement Register (KELA). Pharmacy claims data were also used in the identification of hypertension, dyslipidaemia and diabetes. The definitions of comorbidity groups, along with their corresponding ICD‐10, reimbursement and ATC codes, are provided in Table S1.

### 2.2 Comorbidity Groups

To assess the burden of multimorbidity, we utilised previously established disease‐group definitions developed for Finnish healthcare registry data. Using disease groups captures the full clinical spectrum recorded in electronic health records more comprehensively than solely counting individual diagnosis codes and helps reduce misclassification arising from heterogeneous or overlapping ICD‐10 codes [15, 32]. The original multimorbidity groups were constructed by Wikström et al. using three‐digit ICD‐10 codes and corresponding ICPC‐2 codes with subcodes representing acute manifestations excluded [32], based on a list of chronic conditions previously provided by Calderón‐Larrañaga [33]. Detailed information on the development, organisation and validation of these groups is available in the original publication. We further complemented the comorbidity groups using medication reimbursement data from the Social Insurance Institute and added an additional group representing periodontal diseases. For hypertensive diseases, diabetes and dyslipidaemia, we also supplemented the definitions with pharmacy claims data, as it has been previously shown that these conditions are not reliably captured using diagnosis codes alone [34]. Importantly, a 5‐year look‐back period was applied to define the presence of diagnosis codes in the registers, allowing for accurate evaluation of temporal trends in multimorbidity. The definitions of the codes used in each of the 35 comorbidity groups are presented in Table S2. The number of these groups was then counted for each patient with new‐onset AF.

In addition to the comorbidity groups, we evaluated the prevalence of common comorbidities in patients with AF, including those incorporated in the CHA_2_DS_2_VA/CHA_2_DS_2_VASC risk score. Consistent with the definitions of the comorbidity groups, the 5‐year look‐back period was applied to both the assessment of individual conditions and the calculation of risk scores.

### 2.3 Study Ethics

The study protocol was approved by the Ethics Committee of the Medical Faculty of Helsinki University, Helsinki, Finland (nr. 15/2017 and 15/2024) and received research permission from the Helsinki University Hospital (HUS/46/2018, HUS/217/2024). Respective permissions were obtained from the Finnish register holders (KELA 138/522/2018, THL 2101/5.05.00/2018, Population Register Centre VRK/1291/2019‐3, Statistics Finland TK‐53‐1713‐18/u1281 and Tax Register VH/874/07.01.03/2019). Patients' personal identification numbers were pseudonymised, and the research group received individualised but unidentifiable data. Informed consent was waived due to the retrospective registry nature of the study. The study conforms to the Declaration of Helsinki as revised in 2024.

### 2.4 Statistical Analysis

Descriptive statistics and comorbidity groups by entry‐year are presented as means (continuous variables) or proportions (categoric variables). Trends in categorical variables were assessed using the chi‐square test for linear trend, and trends in continuous variables were evaluated using a one‐way analysis of variance test for linearity. Linear regression was used to assess the association between cohort entry year and the number of comorbidity groups. Three models were fitted: an unadjusted model, a model adjusted for age using a natural cubic spline with three degrees of freedom, and a model additionally adjusted for sex. Regression coefficients with 95% confidence intervals (CIs) were reported. Statistical analyses were conducted using IBM SPSS Statistics software version 28.0 (SPSS Inc., Chicago, Illinois, USA) and R version 4.0.5 (R Core Team, Vienna, Austria; https://www.R‐project.org).

## 3 Results

A total of 196,536 patients (mean age was 72.9 (SD 13.1) years; 49.9% female) with new‐onset AF were identified between 2009 and 2018. Over the study period, the mean age at diagnosis of new‐onset AF increased from 71.8 to 74.0 years. Men were younger than women at diagnosis (mean age 69.2 vs. 77.3 years, *p* < 0.001). Patient characteristics at AF diagnosis, stratified by year of AF diagnosis, are presented in Table 1.

**TABLE 1:** Baseline characteristics of the study cohort according to the year of AF diagnosis.

Entry year (*n*)	2009 (16,222)	2010 (16,259)	2011 (18,806)	2012 (19,228)	2013 (19,787)	2014 (19,799)	2015 (20,053)	2016 (21,599)	2017 (22,127)	2018 (22,656)	All (196,536)
Women	51.0%	51.1%	50.1%	50.0%	49.7%	50.4%	50.0%	49.3%	49.3%	49.0%	49.9% *p* < 0.001
Age, years (mean/SD)	71.8 (13.9)	71.8 (13.9)	72.4 (13.4)	72.6 (13.2)	72.5 (13.3)	72.9 (13.0)	73.2 (12.8)	73.5 (12.6)	73.7 (12.5)	74.0 (12.3)	72.9 (13.1) *p* < 0.001
CHA_2_DS_2_VA (mean/SD)	2.9 (1.7)	2.9 (1.7)	2.9 (1.6)	2.9 (1.6)	2.9 (1.6)	2.9 (1.6)	3.0 (1.6)	2.9 (1.5)	3.0 (1.5)	3.0 (1.5)	2.9 (1.6) *p* < 0.001
CHA_2_DS_2_VASC (mean/SD)	3.4 (1.8)	3.4 (1.8)	3.4 (1.8)	3.4 (1.8)	3.4 (1.7)	3.4 (1.7)	3.5 (1.7)	3.4 (1.7)	3.4 (1.7)	3.5 (1.7)	3.4 (1.7) *p* < 0.001
Diabetes mellitus	27.5%	28.6%	27.9%	28.8%	29.0%	29.1%	29.7%	29.8%	29.4%	29.4%	29.0% *p* < 0.001
Hypertension	77.0%	77.8%	79.5%	80.3%	80.4%	80.5%	80.9%	81.0%	81.7%	82.6%	80.3% *p* < 0.001
Heart failure	18.5%	18.7%	16.4%	15.8%	15.2%	15.2%	14.5%	13.7%	13.2%	13.6%	15.3% *p* < 0.001
Vascular disease	27.3%	25.6%	25.1%	26.4%	25.8%	26.5%	25.8%	24.9%	25.5%	24.8%	25.7% *p* < 0.001
Stroke	9.3%	9.3%	8.8%	9.1%	9.0%	9.1%	9.2%	8.7%	8.6%	8.8%	9.0% *p* = 0.014
Dyslipidaemia	40.7%	41.4%	44.0%	45.6%	46.2%	47.3%	47.4%	46.8%	48.2%	49.3%	46.0% *p* < 0.001
Abnormal renal function	1.7%	1.7%	1.9%	2.1%	2.0%	2.2%	2.2%	2.1%	2.4%	2.3%	2.1% *p* < 0.001
Abnormal liver function	0.3%	0.3%	0.3%	0.3%	0.3%	0.3%	0.4%	0.5%	0.4%	0.4%	0.4% *p* = 0.003
Alcohol use disorder	3.2%	3.4%	3.1%	3.0%	3.1%	3.2%	3.0%	2.9%	2.7%	3.0%	3.0% *p* = 0.001
Dementia	5.8%	5.6%	5.5%	5.4%	5.1%	5.5%	5.2%	4.9%	5.7%	5.3%	5.4% *p* = 0.038
Cancer	12.1%	12.4%	12.8%	12.7%	13.4%	13.7%	14.0%	13.8%	14.4%	15.1%	13.5% *p* < 0.001

*Note:* Values denote proportions (%) or mean (SD) when specified. The linearity of proportion trends was tested with Linear‐by‐Linear Association. One‐Way ANOVA was used in mean trends and weighed *p* values are presented. *p* values denote linear change.

At the time of AF diagnosis, the patients had a mean of 4.1 (SD 2.5) comorbidity groups, with women exhibiting more groups than men (4.2 vs. 3.9, *p* < 0.001). Overall, 85.1% of individuals with new‐onset AF had diagnoses spanning two or more comorbidity groups and 28% from six or more groups. The number of comorbidity groups increased with age (Table 2). Most common comorbidity groups at AF onset in the overall cohort were hypertensive diseases (80.5%), obesity and other metabolic diseases (48.5%), diabetes (29.0%), other diseases of the heart and pulmonary circulation (28.0%), chronic eye diseases and blindness (22.6%) and ischaemic heart diseases (20.9%) (Table S3).

**TABLE 2:** Mean number (standard deviation) of disease groups and stroke risk scores by age group and sex.

	Number of comorbidity groups	CHA_2_DS_2_VA score	CHA_2_DS_2_VASC score
Age group (years)			
20–39	1.6 (1.7)	0.6 (0.7)	0.8 (0.9)
40–59	2.9 (2.3)	1.2 (1.0)	1.4 (1.1)
60–79	4.0 (2.5)	2.8 (1.4)	3.2 (1.5)
80+	4.8 (2.4)	4.0 (1.2)	4.7 (1.3)
Sex			
Men	3.9 (2.6)	2.6 (1.6)	2.6 (1.6)
Women	4.2 (2.4)	3.2 (1.5)	4.2 (1.5)

*Note:* All differences < 0.01.

In men, the most frequent comorbidity groups were hypertensive diseases (75.9%), obesity and other metabolic diseases (47.4%), diabetes (28.6%), other diseases of the heart and pulmonary circulation (26.6%), ischaemic heart diseases (22.3%) and chronic eye diseases and blindness (17.6%). Among women, the most frequent comorbidity groups were hypertensive diseases (85.1%), obesity and other metabolic diseases (49.6%), other diseases of the heart and pulmonary circulation (29.6%), diabetes (29.4%), chronic eye diseases and blindness (27.6%) and ischaemic heart diseases (19.5%) (Table S4).

Age at AF diagnosis increased over the study period in both sexes, rising from 67.3 to 70.9 years in men and from 76.1 to 77.3 years in women (both *p* < 0.01). Also, the prevalence of hypertension, diabetes, dyslipidaemia, abnormal renal or liver function, excess alcohol consumption and cancer increased. In contrast, the prevalence of heart failure, vascular disease and dementia declined, while the prevalence of prior stroke at AF diagnosis remained stable. Consistent with these trends, CHA_2_DS_2_VA and CHA_2_DS_2_VASC scores also increased over time (Table 1).

**FIGURE 1:**
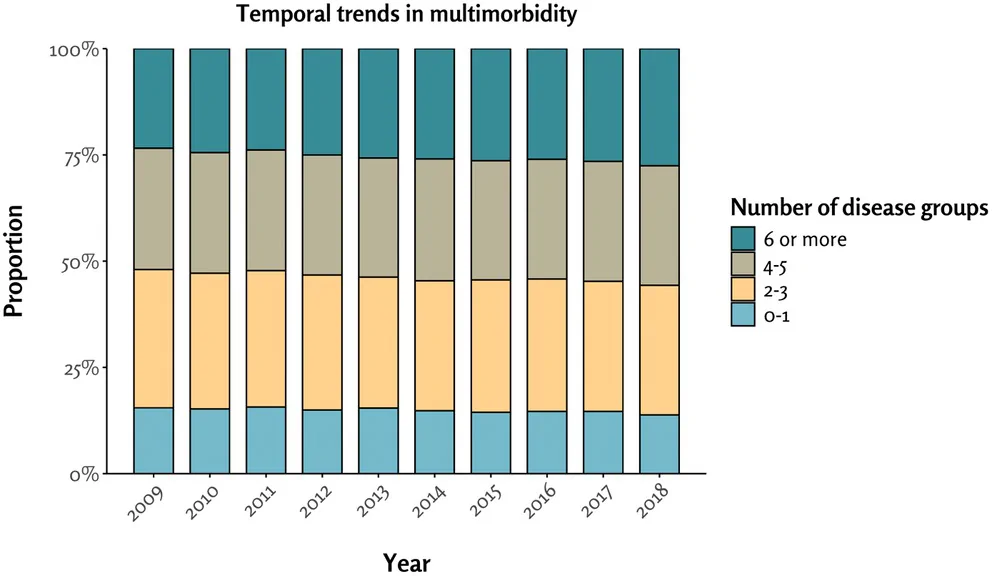
Distribution of the number of comorbidity groups according to the year of incident atrial fibrillation diagnosis. The mean number of comorbidity groups increased over time (unadjusted linear regression: *β* = 0.030 per year, 95% CI 0.026–0.034, *p* < 0.001). The association remained significant after adjustment for age using a natural cubic spline with three degrees of freedom (*β* = 0.014, 95% CI 0.011–0.018, *p* < 0.001) and after additional adjustment for sex (*β* = 0.014, 95% CI 0.010–0.018, *p* < 0.001).

**FIGURE 2:**
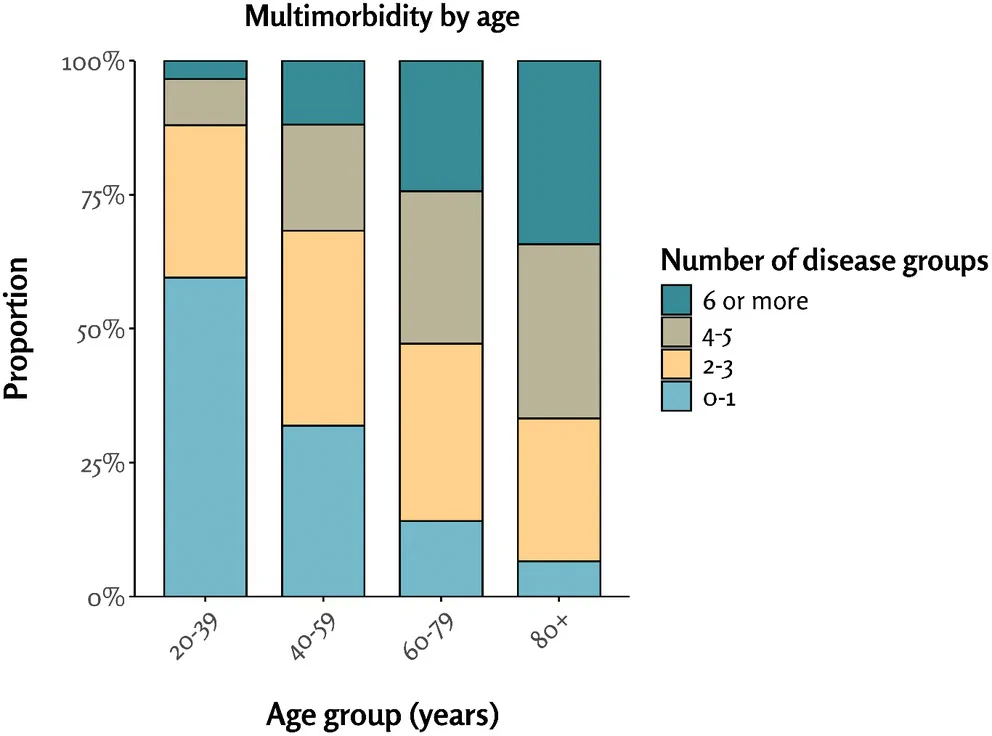
Number of disease groups by age at AF diagnosis.

During the study period, the mean number of comorbidity groups at AF onset increased from 3.9 (SD 2.4) to 4.2 (SD 2.6) (Figure 1). Stratified by sex, the mean number rose from 3.7 (SD 2.4) to 4.1 (SD 2.6) in men and from 4.1 (SD 2.3) to 4.3 (SD 2.5) in women (both *p* < 0.01). Mean numbers of comorbidity groups, along with CHA_2_DS_2_VA/CHA_2_DS_2_VASC scores by age group and sex, are presented in Table 2 and Figure 2.

Later cohort entry year was associated with a higher number of comorbidity groups in the unadjusted analysis (*β* = 0.030 per year, 95% CI 0.026–0.034, *p* < 0.001). After adjustment for age using a natural cubic spline with three degrees of freedom, the association remained significant but was attenuated (*β* = 0.014, 95% CI 0.011–0.018, *p* < 0.001). Additional adjustment for sex had minimal impact on the estimate (*β* = 0.014, 95% CI 0.010–0.018, *p* < 0.001).

The largest relative increases in individual groups were observed for sleep disorders (+138%), periodontal diseases (+75%) and endocrine diseases (+39%). Hypertensive diseases and diabetes also showed increases, each rising by 7%. In contrast, the largest decreases occurred in ischaemic heart diseases (−26%), diseases of the oesophagus, stomach and duodenum (−17%) and other diseases of the heart and pulmonary circulation (−10%).

When examined by sex, the prevalence of diabetes increased by 15% in men, whereas no change was observed in women. In contrast, the prevalence of ischaemic heart disease declined by 16% in men and 38% in women. Comorbidity groups are presented in more detail in Tables S3 and S4.

## 4 Discussion

This nationwide cohort study investigated the prevalence and temporal trends of multimorbidity in patients with AF, with the following principal findings. First, multimorbidity was highly prevalent and increasing over time among patients with AF, with 85.1% of individuals with new‐onset AF having diagnoses from at least two comorbidity groups. Second, multimorbidity was particularly common among women and older patients. Third, although the prevalence of most comorbidity groups increased over time, the prevalence of ischaemic heart disease and other diseases of the heart and pulmonary circulation declined.

The observed increase in multimorbidity over time was partly explained by differences in age distribution between cohorts, as adjustment for age attenuated the association between cohort entry year and the number of comorbidity groups. Nevertheless, the association remained significant after adjustment for age and sex, indicating that the temporal increase in multimorbidity was not fully explained by demographic changes in the study population.

The most common comorbidity groups were hypertensive diseases (≈80%), obesity and other metabolic diseases (≈50%), diabetes (≈30%) and other cardiovascular diseases. Consistent with the overall trends in comorbidity groups and individual diseases, stroke risk scores (CHA_2_DS_2_VA and CHA_2_DS_2_VASC) increased over time. The prevalence of prior stroke at the time of AF diagnosis did not change over the study period.

A high prevalence of multimorbidity among patients with AF has been reported in prior studies [38, 39]. However, heterogeneity in the definitions of multimorbidity complicates the assessment of temporal trends and comparisons between cohorts. One study from the UK reported a rising comorbidity burden at AF diagnosis between 1998 and 2017, with the proportion of patients having three or more comorbidities increasing from 48% to 68%, accompanied by a declining prevalence of ischaemic heart disease and heart failure—trends consistent with our findings [35]. However, whereas the UK study examined 18 predefined diagnoses, the present study encompasses the entire spectrum of diagnostic codes, with comorbidity groups derived from hundreds of diagnoses, thereby capturing a broader range of conditions and more comprehensively reflecting the complexity of multimorbidity. Our findings of a stable prevalence of prior stroke at AF onset, together with a decrease in ischaemic heart disease, are consistent with previous literature [36].

Our results expand previous knowledge of the clinical complexity of patients with AF. The observed increase in multimorbidity parallels the increasing age of the patients with AF, and this trend is likely to continue as the population further ages. Concurrently, the incidence of AF is also increasing, underscoring the importance of addressing the risk factors and comorbidities associated with AF. Targeting these risk factors is essential not only in individuals without AF to prevent its development, but also in those with AF, to optimise clinical outcomes and reduce the burden of AF [35]. The most prevalent comorbidity groups observed in patients with AF, including hypertension, obesity and other metabolic disorders, are well‐established and treatable risk factors for stroke in patients with AF. The increasing multimorbidity burden in patients with AF calls for holistic or integrated care management approaches [37], which have been shown to be associated with improved clinical outcomes [38] and are strongly recommended in guidelines and patient care pathways worldwide [39, 40].

### 4.1 Limitations

The inherent limitations of a retrospective, registry‐based study design should be acknowledged. Possible inaccuracies in administrative data have the potential of introducing information bias. Part of the observed trend in multimorbidity may also reflect the changes in diagnostic awareness, screening practices, healthcare utilisation or coding practices, rather than true increases in disease prevalence. However, we sought to mitigate these limitations by collecting data from multiple nationwide registers, including reimbursement and pharmacy claims, and by applying a long, fixed look‐back period of 5 years. The fixed look‐back period also enabled the evaluation of temporal trends while mitigating the potential impact of variations in recording practices. Moreover, the Finnish national registers have been evaluated by the World Bank as having the highest‐performing administrative statistical system worldwide [41]. The national specialist care register is well‐validated and demonstrates high accuracy, particularly for cardiovascular diseases [42, 43]. Finally, additional studies in different populations are warranted given the reported racial differences in clinical epidemiology and cardiovascular conditions [44, 45]. Another limitation is the absence of an age‐matched non‐AF control group. Future studies are needed to determine whether the increase in multimorbidity is specific to patients with AF or if it represents a general trend across the population.

## 5 Conclusions

In conclusion, this comprehensive nationwide study demonstrates that multimorbidity is highly prevalent and increasing among patients with AF. These findings highlight the substantial burden of multimorbidity and support the recommendations of current clinical guidelines for a holistic management approach for patients with AF.

## Author Contributions

MD Reinilä had full access to all the data in the study and takes responsibility for the integrity of the data and the accuracy of the data analysis. Concept and design: Reinilä, Langén, Jaakkola, Teppo. Acquisition, analysis or interpretation of data: All authors. Drafting of the manuscript: Reinilä. Critical revision of the manuscript for important intellectual content: All authors. Statistical analysis: Reinilä, Teppo. Obtained funding: Teppo. Administrative, technical or material support: Jaakkola, Halminen. Supervision: Langén, Jaakkola, Teppo, Putaala, Mustonen, Airaksinen, Lehto.

## Funding

This work was supported by the Aarne Koskelo Foundation, The Finnish Foundation for Cardiovascular Research, and Helsinki and Uusimaa Hospital District Research Fund (TYH2019309).

## Conflicts of Interest

Sanni Reinilä: none. Niklas Kiuru: none. Konsta Teppo: Research Grants: The Finnish Foundation for Cardiovascular Research, Aarne and Aili Turunen Foundation, The Finnish Medical Foundation, The Finnish Foundation for Alcohol Studies and the Finnish State Research Funding. Jussi Jaakkola: none. Ville Langén: Research grants: The State Research Funding of the wellbeing services county of Southwest Finland; Speaker: Boehringer‐Ingelheim. Olli Halminen: none. Jukka Putaala: Speaker: Bayer, Boehringer‐Ingelheim, BMS‐Pfizer, Abbott; Advisory board: Portola, Novo Nordisk, Herantis Pharma; Visiting editor: Terve Media; Stock ownership: Vital Signum. Ossi Lehtonen: none. Pirjo Mustonen: Consultant: Roche, BMS‐Pfizer‐alliance, Novartis Finland, Boehringer Ingelheim, MSD Finland. Jari Haukka: Consultant: Research Janssen R&D; Speaker: Bayer Finland. Miika Linna: Speaker: BMS‐Pfizer‐alliance, Bayer, Boehringer‐Ingelheim. Juha Hartikainen: Research grants: The Finnish Foundation for Cardiovascular Research, EU Horizon 2020, EU FP7. Advisory Board Member: BMS‐Pfizer‐alliance, Novo Nordisk, Amgen. Speaker: Cardiome, Bayer. K.E. Juhani Airaksinen: Research grants: The Finnish Foundation for Cardiovascular Research; Speaker: Bayer, Boehringer‐Ingelheim. Mika Lehto: Consultant: BMS‐Pfizer‐alliance, Bayer, Boehringer‐Ingelheim and Organon; Speaker: BMS‐Pfizer‐alliance, Bayer, Boehringer Ingelheim and Organon. Research grants: Aarne Koskelo Foundation, The Finnish Foundation for Cardiovascular Research, and Helsinki and Uusimaa Hospital District research fund. Gregory Y. H. Lip reports institutional consultancy and/or speaker roles for BMS/Pfizer, Boehringer Ingelheim, Anthos, Huawei (No fees are received personally). He is co‐PI of the AFFIRMO project on multimorbidity in AF (grant agreement No 899871), TARGET project on digital twins for personalised management of atrial fibrillation and stroke (grant agreement No 101136244) and ARISTOTELES project on artificial intelligence for management of chronic long term conditions (grant agreement No 101080189), which are all funded by the EU's Horizon Europe Research & Innovation programme.

## Supporting information


**Table S1:** Definitions of comorbidities.
**Table S2:** Definitions of comorbidity groups.
**Table S3:** The trends of comorbidity groups.
**Table S4:** The trends of comorbidity groups by sex.

## Data Availability

The data that support the findings of this study are available from Social and Health Data Permit Authority. Restrictions apply to the availability of these data, which were used under licence for this study. Data are available from https://findata.fi/en/ with the permission of Social and Health Data Permit Authority.
