# Supplementary material for: Temporal Trends in Multimorbidity Patterns in Patients With Atrial Fibrillation—A Nationwide Cohort Study
Source: Eur J Clin Invest. 2026 Aug 21;56(8):e70253. doi: 10.1111/eci.70253 (PMC13498663; doi:10.1111/eci.70253)
Supplement: Supplementary file 1 [file ECI-56-e70253-s001.docx]

**Supplementary File**

**Supplementary Table 1.** Definitions of comorbidities.

**Supplementary Table 2.** Definitions of comorbidity groups.

**Supplementary Table 3.** The trends of comorbidity groups

**Supplementary Table 4.** The trends of comorbidity groups by sex

**Supplementary Table 1.** Definitions of comorbidities.

|  | ICD-10 | Reimbrusement code | ATC code | Other |
| --- | --- | --- | --- | --- |
| Diabetes | E10–14 | 103, 215, 285 | A10 |  |
| Hypertension | I10–I15 | 205 | C03A, C03B, C03DB, C03EA, C07A, C08CA, C08D, C09 |  |
| Any vascular disease | I20–I25, I65–I66, I67.2, I70 | 206 |  |  |
| Heart Failure | I50, I11.0, I13.0, I13.2 | 201 |  |  |
| Alcohol abuse | F10 |  |  |  |
| Cancer |  |  |  | Any cancer registered in the FinnishCancer Registry |
| Abnormal renal function | N18, Z49 |  |  |  |
| Abnormal liver function | K70.2–K70.4, K71.7, K71.8, K72, K74 |  |  |  |
| Dementia | F00–F03, G30 |  |  |  |
| History of stroke | I63, I64, I69.3–I69.8 |  |  |  |
| Dyslipidaemia | E78 | 206 | C10 |  |
| Abbreviations: ATC, anatomic therapeutic chemical; ICD-10, International Classification of Diseases, 10th Revision | | | | |

**Supplementary Table 2.** Definitions of comorbidity groups.

|  | ICD-10 | Reimbrusement code | ATC code |
| --- | --- | --- | --- |
| Cancer and in-situ carcinomas | C01–26, C31–34, C37–41, C43–58, C60–85, C88, C90–97, D00–07, D09 | 115–117, 128, 130 |  |
| Chronic skin diseases | L10, L12–13, L20–21, L23–24, L26, L28, L40–41, L43–45 | 134, 319, 380 |  |
| Inflammatory diseases of joints and connective tissue | M05–14, M30–36 | 202, 212, 313 |  |
| Arthrosis | M15–19 |  |  |
| Other musculoskeletal diseases | M20–24, M70, M72–73, M75, M79–84, M91, M93–94, M96, M99 |  |  |
| Back diseases | M40–43, M45–51, M53–54 |  |  |
| Chronic diseases of the kidneys and urinary tract | N01, N03–05, N07–08, N11, N18–22, N31–33, N35 | 137–138 |  |
| Diseases of male genitals/reproductive organs | N40–42, N48, N50 |  |  |
| Diseases of female genitals/reproductive organs | N80–81, N88 |  |  |
| Blood diseases and blood-forming organs | D50–53, D55–61, D63–64, D66–69, D71–77, D80–84, D86, D89 | 122, 126, 129 |  |
| Endocrine diseases | E00–03, E05–07, E20–29, E31, E34–35 | 101–103, 105–106, 121, 123–124, 186 |  |
| Diabetes | E10–14 | 103, 215, 285 | A10 |
| Obesity and other metabolic diseases | E65–66, E68, E70–72, E74–80, E83–85, E88–90 | 254, 292, 294, 388, 3015, 3051 | C10 |
| Dementia and organic psychical disorders | F00–07, F09, G30–32 |  |  |
| Psychiatric and behavioural diseases related to substance abuse | F10–16, G18–19 |  |  |
| Schizophrenia and delusional diseases | F20–22, F24–25, F28–29 | 112 |  |
| Mood disorders | F30–34, F38–39 |  |  |
| Neurotic, stress-related and somatoform diseases, including eating disorders | F40–42, F44–45, F48, F50, F52–55, F59–63, F68–69 |  |  |
| Sleep disorders | F51, G47 |  |  |
| Other neurological diseases | G10–13, G20–26, G35, G37 | 109–110, 119, 214 |  |
| Epilepsy and migraine | G40–41, G43–44 | 111 |  |
| Diseases of nerves and nerve-muscle junction | G50–60, G62–64, G70–73 | 108 |  |
| Chronic eye diseases and blindness | H17–21, H25–28, H31, H33, H35–36, H40, H42, H47–49, H51, H54 | 114 |  |
| Chronic ear diseases and deafness | H80–83, H90–91, H93, H95 |  |  |
| Hypertensive diseases | I10–13, I15, I95, I97–99 | 205 | C03A, C03B, C03DB, C03EA, C07A, C08CA, C08D, C09 |
| Ischemic heart diseases | I20–25 | 206, 287, 296, 386 |  |
| Other diseases of the heart and pulmonary circulation | I27–28, I31, I34–39, I42–45, I49–51 | 201, 203, 207, 250, 290, 342, 351, 379 |  |
| Cerebrovascular diseases | I60–69 |  |  |
| Diseases or arteries and veins | I70–73, I78–79, I83–87, I89 |  |  |
| Chronic diseases of the upper respiratory tract | J30–35, J37–38 |  |  |
| Chronic diseases of the lower respiratory tract | J41–47  J60–68, J70, J84, J92, J95-96, J98–99 |  |  |
| Periodontal diseases | K05 |  |  |
| Diseases of the oesophagus, stomach, and duodenum | K21–23, K25–31 |  |  |
| Inflammatory and other bowel diseases | K50–52, K55, K57–59, K62–63, K90 | 208, 326 |  |
| Diseases of the liver, pancreas, and biliary tract | K70–77, K80–81, K86 | 133 |  |

**Supplementary Table 3.** The trends of comorbidity groups

|  | All (%)  (N = 196 536) | 2009 (%)  (N = 24449) | 2018 (%)  (N = 25409) | Change  (%) | p-value (Linear-by-Linear) |
| --- | --- | --- | --- | --- | --- |
| Cancer and in-situ carcinomas | 13.9 | 12.2 | 15.5 | 27 | <0.001 |
| Chronic skin diseases | 2.2 | 1.8 | 2.3 | 28 | <0.001 |
| Inflammatory diseases of joints and connective tissue | 5.9 | 5.8 | 5.8 | 0 | 0.043 |
| Arthrosis | 12.8 | 11.9 | 13.6 | 14 | <0.001 |
| Other musculoskeletal diseases | 12.1 | 11.4 | 12.4 | 9 | 0.002 |
| Back diseases | 9.7 | 8.8 | 10.6 | 20 | <0.001 |
| Chronic diseases of the kidneys and urinary tract | 5.0 | 4.4 | 5.8 | 32 | <0.001 |
| Diseases of male genitals or reproductive organs | 5.5 | 5.0 | 5.8 | 16 | <0.001 |
| Diseases of female genitals/reproductive organs | 1.5 | 1.5 | 1.4 | -7 | 0.199 |
| Blood diseases and blood-forming organs | 6.1 | 6.0 | 6.0 | 0 | 0.099 |
| Endocrine diseases | 9.5 | 7.1 | 9.9 | 39 | <0.001 |
| Diabetes | 29.0 | 27.5 | 29.4 | 7 | <0.001 |
| Obesity and other metabolic diseases | 48.5 | 42.9 | 52.1 | 21 | <0.001 |
| Dementia and organic psychical disorders | 6.4 | 6.6 | 6.5 | -2 | 0.284 |
| Psychiatric and behavioural diseases related to substance abuse | 3.2 | 3.3 | 3.2 | -3 | 0.009 |
| Schizophrenia and delusional diseases | 1.1 | 1.1 | 1.1 | 0 | 0.701 |
| Mood disorders | 2.7 | 2.9 | 2.6 | -10 | <0.001 |
| Neurotic, stress-related and somatoform diseases, including eating disorders | 1.7 | 1.7 | 1.8 | 6 | 0.926 |
| Sleep disorders | 3.5 | 2.1 | 5.0 | 138 | <0.001 |
| Other neurological diseases | 1.8 | 1.7 | 2.0 | 18 | 0.001 |
| Epilepsy and migraine | 2.8 | 2.8 | 2.9 | 4 | 0.641 |
| Diseases of nerves and nerve-muscle junction | 4.6 | 4.2 | 4.9 | 17 | <0.001 |
| Chronic eye diseases and blindness | 22.6 | 20.9 | 24.3 | 16 | <0.001 |
| Chronic ear diseases and deafness | 10.8 | 9.8 | 11.4 | 16 | <0.001 |
| Hypertensive diseases | 80.5 | 77.1 | 82.7 | 7 | <0.001 |
| Ischemic heart diseases | 20.9 | 25.4 | 18.7 | -26 | <0.001 |
| Other diseases of the heart and pulmonary circulation | 28.0 | 30.4 | 27.5 | -10 | <0.001 |
| Cerebrovascular diseases | 11.4 | 11.6 | 11.3 | -3 | 0.155 |
| Diseases or arteries and veins | 0.9 | 9.8 | 10.5 | 7 | 0.001 |
| Chronic diseases of the upper respiratory tract | 2.5 | 2.4 | 2.5 | 4 | 0.302 |
| Chronic diseases of the lower respiratory tract | 10.4 | 10.0 | 10.5 | 5 | 0.628 |
| Periodontal diseases | 0.5 | 0.4 | 0.7 | 75 | <0.001 |
| Diseases of the oesophagus, stomach, and duodenum | 5.9 | 6.5 | 5.4 | -17 | <0.001 |
| Inflammatory and other bowel diseases | 9.5 | 9.0 | 10.2 | 13 | <0.001 |
| Diseases of the liver, pancreas, and biliary tract | 4.3 | 4.2 | 4.3 | 2 | 0.734 |
| The linearity of proportion trends was tested with Linear-by-Linear Association. One-Way ANOVA was used in mean trends and weighed p-values are presented. P-values denote significancy for linear change over study period. Only proportions at the start and the end of study period are presented for simplicity. | | | | | |

**Supplementary Table 4.** The trends of comorbidity groups by sex

|  | Sex | All (%) | 2009 (%) | 2018 (%) | Change (%) | p-value  (linear-by-linear) |
| --- | --- | --- | --- | --- | --- | --- |
| Cancer and in-situ carcinomas | Men | 14.6 | 13.0 | 16.6 | 28 | <0.001 |
|  | Women | 13.1 | 11.5 | 14.4 | 25 | <0.001 |
| Chronic skin diseases | Men | 2.2 | 2.1 | 2.5 | 19 | 0.006 |
|  | Women | 2.1 | 1.6 | 2.2 | 38 | <0.001 |
| Inflammatory diseases of joints and connective tissue | Men | 4.9 | 4.5 | 5.3 | 18 | <0.001 |
|  | Women | 6.9 | 7.1 | 6.4 | -10 | 0.274 |
| Arthrosis | Men | 10.1 | 9.2 | 10.6 | 15 | <0.001 |
|  | Women | 15.6 | 14.4 | 16.7 | 16 | <0.001 |
| Other musculoskeletal diseases | Men | 10.2 | 10.0 | 10.1 | 1 | 0.390 |
|  | Women | 14.1 | 12.8 | 14.8 | 16 | <0.001 |
| Back diseases | Men | 8.4 | 7.8 | 8.9 | 14 | <0.001 |
|  | Women | 11.0 | 9.7 | 12.3 | 27 | <0.001 |
| Chronic diseases of the kidneys and urinary tract | Men | 6.2 | 5.3 | 7.2 | 36 | <0.001 |
|  | Women | 3.9 | 3.4 | 4.4 | 29 | <0.001 |
| Diseases of male genitals/reproductive organs | Men | 11.1 | 10.2 | 11.4 | 12 | <0.001 |
|  | Women | - | - | - | - | - |
| Diseases of female genitals/reproductive organs | Men | - | - | - | - | - |
|  | Women | 3.0 | 3.0 | 2.9 | -3 | 0.510 |
| Blood diseases and blood-forming organs | Men | 5.4 | 5.4 | 5.6 | 4 | 0.116 |
|  | Women | 6.7 | 6.6 | 6.4 | -3 | 0.320 |
| Endocrine diseases | Men | 7.9 | 5.9 | 8.1 | 37 | <0.001 |
|  | Women | 11.2 | 8.3 | 11.8 | 42 | <0.001 |
| Diabetes | Men | 28.6 | 25.8 | 29.7 | 15 | <0.001 |
|  | Women | 29.4 | 29.1 | 29.0 | 0 | 0.965 |
| Obesity and other metabolic diseases | Men | 47.4 | 41.8 | 51.6 | 23% | <0.001 |
|  | Women | 49.6 | 44.6 | 52.5 | 18 | <0.001 |
| Dementia and organic psychical disorders | Men | 4.8 | 4.7 | 4.9 | 4 | 0.547 |
|  | Women | 8.0 | 8.4 | 8.1 | -4 | 0.152 |
| Psychiatric and behavioural diseases related to substance abuse | Men | 5.1 | 5.5 | 4.8 | -13 | <0.001 |
|  | Women | 1.3 | 1.2 | 1.5 | 25 | 0.035 |
| Schizophrenia and delusional diseases | Men | 0.9 | 0.9 | 0.8 | -11 | 0.192 |
|  | Women | 1.3 | 1.3 | 1.3 | 0 | 0.476 |
| Mood disorders | Men | 2.3 | 2.4 | 2.0 | -17 | 0.004 |
|  | Women | 3.1 | 3.4 | 3.1 | -9 | 0.079 |
| Neurotic, stress-related and somatoform diseases, including eating disorders | Men | 1.3 | 1.4 | 1.3 | -7 | 0.051 |
|  | Women | 2.0 | 1.9 | 2.3 | 21 | 0.097 |
| Sleep disorders | Men | 5.0 | 3.3 | 6.7 | 103 | <0.001 |
|  | Women | 2.0 | 1.0 | 3.4 | 240 | <0.001 |
| Other neurological diseases | Men | 1.8 | 1.7 | 2.0 | 18 | 0.005 |
|  | Women | 1.9 | 1.7 | 2.1 | 24 | 0.081 |
| Epilepsy and migraine | Men | 2.4 | 2.6 | 2.6 | 0 | 0.422 |
|  | Women | 3.2 | 3.1 | 3.4 | 10 | 0.138 |
| Diseases of nerves and nerve-muscle junction | Men | 4.5 | 4.0 | 4.9 | 23 | <0.001 |
|  | Women | 4.7 | 4.3 | 4.9 | 14 | 0.004 |
| Chronic eye diseases and blindness | Men | 17.6 | 15.1 | 20.0 | 32 | <0.001 |
|  | Women | 27.6 | 26.5 | 28.7 | 8 | <0.001 |
| Chronic ear diseases and deafness | Men | 9.6 | 7.9 | 10.1 | 28 | <0.001 |
|  | Women | 12.1 | 11.5 | 12.7 | 10 | <0.001 |
| Hypertensive diseases | Men | 75.9 | 70.4 | 79.5 | 13 | <0.001 |
|  | Women | 85.1 | 83.6 | 86.2 | 3 | <0.001 |
| Ischemic heart diseases | Men | 22.3 | 25.2 | 21.2 | -16 | <0.001 |
|  | Women | 19.5 | 25.6 | 16.0 | -38 | <0.001 |
| Other diseases of the heart and pulmonary circulation | Men | 26.6 | 27.8 | 26.0 | -6 | 0.005 |
|  | Women | 29.6 | 33.0 | 28.4 | -14 | <0.001 |
| Cerebrovascular diseases | Men | 10.8 | 10.3 | 11.0 | 7 | 0.426 |
|  | Women | 12.0 | 12.9 | 11.6 | -10 | 0.009 |
| Diseases or arteries and veins | Men | 9.9 | 9.7 | 11.0 | 13 | <0.001 |
|  | Women | 9.9 | 10.0 | 10.0 | 0 | 0.465 |
| Chronic diseases of the upper respiratory tract | Men | 2.7 | 2.8 | 2.7 | -4 | 0.024 |
|  | Women | 2.2 | 2.0 | 2.2 | 10 | 0.400 |
| Chronic diseases of the lower respiratory tract | Men | 10.8 | 10.5 | 10.8 | 3 | 0.138 |
|  | Women | 10.0 | 9.5 | 10.2 | 7 | 0.033 |
| Periodontal diseases | Men | 0.6 | 0.4 | 0.8 | 100 | <0.001 |
|  | Women | 0.3 | 0.3 | 0.6 | 100 | <0.001 |
| Diseases of the oesophagus, stomach, and duodenum | Men | 5.5 | 6.0 | 5.2 | -13 | <0.001 |
|  | Women | 6.2 | 6.9 | 5.7 | -17 | <0.001 |
| Inflammatory and other bowel diseases | Men | 8.1 | 7.8 | 8.6 | 10 | <0.001 |
|  | Women | 11.0 | 10.2 | 11.8 | 16 | <0.001 |
| Diseases of the liver, pancreas, and biliary tract | Men | 4.3 | 4.1 | 4.2 | 2 | 0.861 |
|  | Women | 4.3 | 4.3 | 4.3 | 0 | 0.518 |
| The linearity of proportion trends was tested with Linear-by-Linear Association. One-Way ANOVA was used in mean trends and weighed p-values are presented. P-values denote significancy for linear change over study period. Only proportions at the start and the end of study period are presented for simplicity. | | | | | | |
